# α7 Nicotinic Receptor-Mediated Astrocytic Gliotransmitter Release: Aβ Effects in a Preclinical Alzheimer’s Mouse Model

**DOI:** 10.1371/journal.pone.0081828

**Published:** 2013-11-28

**Authors:** Tiina Maria Pirttimaki, Neela Krushna Codadu, Alia Awni, Pandey Pratik, David Andrew Nagel, Eric James Hill, Kelly Tennyson Dineley, H. Rheinallt Parri

**Affiliations:** 1 School of Life and Health Sciences, Aston University, Birmingham, England; 2 Aston Research Centre into Healthy Ageing, Aston University, Birmingham, England; 3 Department of Neurology, University of Texas Medical Branch at Galveston, Galveston, Texas, United States of America; Centre national de la recherche scientifique, University of Bordeaux, France

## Abstract

It is now recognized that astrocytes participate in synaptic communication through intimate interactions with neurons. A principal mechanism is through the release of gliotransmitters (GTs) such as ATP, D-serine and most notably, glutamate, in response to astrocytic calcium elevations. We and others have shown that amyloid-β (Aβ), the toxic trigger for Alzheimer’s disease (AD), interacts with hippocampal α7 nicotinic acetylcholine receptors (nAChRs). Since α7nAChRs are highly permeable to calcium and are expressed on hippocampal astrocytes, we investigated whether Aβ could activate astrocytic α7nAChRs in hippocampal slices and induce GT glutamate release. We found that biologically-relevant concentrations of Aβ_1-42_ elicited α7nAChR-dependent calcium elevations in hippocampal CA1 astrocytes and induced NMDAR-mediated slow inward currents (SICs) in CA1 neurons. In the Tg2576 AD mouse model for Aβ over-production and accumulation, we found that spontaneous astrocytic calcium elevations were of higher frequency compared to wildtype (WT). The frequency and kinetic parameters of AD mice SICs indicated enhanced gliotransmission, possibly due to increased endogenous Aβ observed in this model. Activation of α7nAChRs on WT astrocytes increased spontaneous inward currents on pyramidal neurons while α7nAChRs on astrocytes of AD mice were abrogated. These findings suggest that, at an age that far precedes the emergence of cognitive deficits and plaque deposition, this mouse model for AD-like amyloidosis exhibits augmented astrocytic activity and glutamate GT release suggesting possible repercussions for preclinical AD hippocampal neural networks that contribute to subsequent cognitive decline.

## Introduction

It is now recognized that astrocytes contribute significantly to synaptic communication and, in concert with presynaptic and postsynaptic neuronal elements, participate in the underlying mechanisms of brain plasticity, learning and memory. Astrocytes ensheath synapses and modulate synaptic function through the release of gliotransmitters (GTs) such as ATP, D-serine and glutamate [1-3] in response to elevations in astrocytic intracellular Ca^2+^ ([Ca^2+^]_i_) [4]. The interaction between neuronal neurotransmitter receptors and astrocytes [5] and the subsequent release of GTs has been shown to be involved in many processes including mediation of hippocampal long term potentiation [6,7] and learning [8]. 

Modulation of synaptic transmission through astrocytic Ca^2+^-dependent GT release can be induced by neuronal transmitters that act upon astrocytic plasma membrane receptors. One unexplored neuronal signal that could potentially impact astrocyte to neuron communication is the Alzheimer’s disease (AD) toxicant amyloid-beta (Aβ), since it has been demonstrated that synaptic activity leads to increased interstitial Aβ under normal physiological conditions [9]. Given that alpha7 nicotinic acetylcholine receptors (α7nAChRs): 1) bind Aβ and are activated with picomolar affinity [10-13], 2) are ligand gated ionotropic receptors highly permeable to Ca^2+^ [14], and 3) are functionally expressed on hippocampal astrocytes [15,16], we investigated the effects of astrocytic α7nAChRs on [Ca^2+^]_i_ and GT release following exposure to Aβ and α7nAChR-selective ligands, as well as evaluated astrocytic activity in a mouse model for AD-like amyloidosis to investigate astrocytic activity and subsequent GT release at an age that considerably precedes manifestation of cognitive impairment and plaque formation. 

In hippocampal slices from WT rats and mice, Aβ_1-42_ at biologically-relevant concentrations elicited α7nAChR-dependent [Ca^2+^]_i_ elevations in hippocampal astrocytes that varied between CA1 strata and induced GT release. In APP mice, we found that spontaneous astrocytic [Ca^2+^]_i_ elevations were of higher frequency compared to wild type and that there was increased GT glutamate release as measured by NMDAR-mediated slow inward currents (SICs) recorded in hippocampal principal cells. These findings also suggest that, in addition to potential effects of aberrant Aβ production on the fidelity of hippocampal synaptic transmission, a long term consequence of Aβ over-production is perturbation of gliotransmission fidelity. 

## Materials and Methods

All procedures were approved by Aston University Bioethics committee, and carried out in accordance with the UK Animals (Scientific Procedures) Act 1986 and associated procedures.

### Slice preparation

Horizontal slices of hippocampus were prepared from 6-23 day old male Wistar rats. Animals were anaesthetised with isofurane and euthenised by cervical dislocation. After removal, the brain was placed in ice cold modified artificial cerebrospinal fluid (ACSF) of composition (mM) NaCl 126, NaHCO_3_ 26, KCl 1, KH_2_PO_4_ 1.25, MgSO_4_ 5, CaCl_2_ 1 and Glucose 10. Slices were then maintained at room temperature (23-25°C) in this solution for a recovery period of 1 hour before experimental use. 

The same protocols were used for preparation of hippocampal slices from 10-28 day old Tg2576 mice and wild type littermates. Animals were bred by mating heterozygous Tg2576 males with C57Bl6/SJL (F1) females (Jackson Laboratory). 

Tail clips were routinely collected following brain removal and sent for genotyping post experimentally (Transnetyx). 

### Solutions and chemicals

The standard recording ACSF used in this study was (in mM): NaCl 126, NaHCO_3_ 26, KCl 2.5, KH_2_PO_4_ 1.25, MgSO_4_ 1, CaCl_2_ 2 and Glucose 10, unless otherwise stated. As we [17] and others have done previously in attempting to enhance NMDA-R mediated current detection, whole cell voltage clamp (V-Clamp) recordings were conducted in 0-Mg^2+^ at room temperature, unless otherwise stated. Pharmacological compounds were included in the ACSF as stated in text. Chemicals were obtained from Sigma-Aldrich unless otherwise stated. PNU282987, PNU 120596, CGP55845, CPCCOEt, LY34195 and PPADS were obtained from Tocris. D-APV, tetrodotoxin (TTX), SR95531, NBQX, MTEP, methyllycaconitine (MLA) and α-bungarotoxin were obtained from Ascent. Fura-2 AM and Sulforhodamine 101 (SR101) were obtained from Invitrogen. Aβ_1-42_ and Aβ_1-40_ were obtained from Bachem. Aβ_1-42_ stock solutions were prepared according to the methods of Dineley (2001). Briefly, Aβ stock solutions were prepared at 100 µM in 200 mM HEPES pH 8.5, aliquoted and stored frozen at -70°C until further dilution in ACSF for treatments. This preparation yields oligomeric Aβ with the predominant forms being trimer and hexamer [18]. The drug cocktail for isolation of astrocytic mediated effects was composed of (in µM): SR95531 (20), NBQX (20), D-APV (50), MTEP (10), CGP55845 (100), CPCCOEt (100), LY341495 (100) PPADS (100) and TTX (0.1). In this study Aβ application was achieved by including in the perfusate. 

### Electrophysiology

The recording chamber and manipulators were mounted on a moveable top plate platform (MP MTP-01, Scientifica). Patch clamp recordings were made using pipettes (2-4MΩ) containing an internal solution of composition (in mM): KMeSO_4_ 120, HEPES 10, EGTA 0.1, Na_2_ATP 4, GTP 0.5 (Adjusted to pH 7.3-7.4 with KOH, osmolarity 285-290 mOsm). Currents were recorded using a Multiclamp 700B amplifier, digitized with a Digidata 1440A and acquired and analysed using PClamp (Molecular Devices). Voltage clamp recordings were made at -60mV. Cells with ≥ 20% change in access resistance were excluded. SICs were analysed using the Event Detection protocols in the Clampfit routine of PClamp. As in our previous studies, events were accepted as SICs if their amplitude was >20pA and their time to peak (indicated as rise time in figures) was >20ms [17,19,20]. Data was exported to Sigmaplot (Jandel) for further analysis and plotting. 

For ELISA, hippocampus and cortex were dissected from P4 and P14 mice from TG2576 litters. Tissue was weighed and combined with 8 x the mass of ice cold Guanidine HCL (5M)l/Tris HCL(50mM) pH 8.0. Tissue was ground using a hand held homogeniser (Thermofisher Scientific) and stored on ice. The homogenate was then mixed 1:20 with ice cold Dulbecco’s phosphate buffered saline (Thermofisher Scientific) with 5% BSA and 0.03% Tween-20, supplemented with Complete mini protease inhibitor cocktail (Roche). The sample was then centrifuged at 16000 X g for 20 minutes at 4C. The supernatant was carefully removed and placed into a fresh microcentrifuge tube on ice. Samples were analysed using the Human Aβ1-42 ELISA kit (Life Technologies) according to the manufacturer’s instructions.

### Fluorescence imaging

To enable comparisons of activity in different conditions in the same astrocytes we used the ratiometric calcium indicator dye Fura-2. Slices were loaded with Fura-2 AM (Molecular Probes) after a post cutting recovery period of 1 hr. This was done by incubating for ~50 min at 30°C with 5µM of the indicator dye and 0.01% pluronic acid. Under these conditions, astrocytes are preferentially loaded [17]. For astrocytic identification, slices were also loaded with 1µM Sulforhodamine 101, according to *in vitro* methods of Kafitz et al. [21]. The recording chamber and manipulators were mounted on a motorized moveable bridge (Luigs and Neumann) and fluorescence dyes were excited using an Optoscan monochromator system, fitted to a Nikon FN1 upright microscope; filter cubes for selective Fura-2 and SR101 imaging were obtained from Chroma . Images of slice areas of 444µm x 341µm were routinely acquired every 5s with a x20 objective lens (NA=0.8) using an ORCA ER CCD camera (Hamamatsu) and analysed using Simple PCI software (Hamamatsu). Only cells positive for the astrocytic marker SR101 were included in the analyses. 

Ratiometric calcium values over time for specific regions of interest (ROIs) were exported and analysed using Sigmaplot (Systat). The number of events during a recording was determined by identifying events where amplitude exceeded 2 standard deviations of baseline variations. Results are expressed as the mean of the number of events per minute for each astrocyte. For determination of amplitude changes, the absolute ratio increases in the different conditions in the same cells were compared. 

### Statistics

All quantitative data in the text and figures are presented as mean±s.e.m. unless otherwise stated. Significance was calculated using multivariate ANOVA and unpaired or paired Student’s *t*-test as appropriate. Kolmogorov-Smirnov tests were used for population distribution comparisons. Statistical significance in figures is indicated as: *P*<0.05 *, *P*<0.01 ** or *P*<0.005 ***. 

## Results

**Figure 1:**
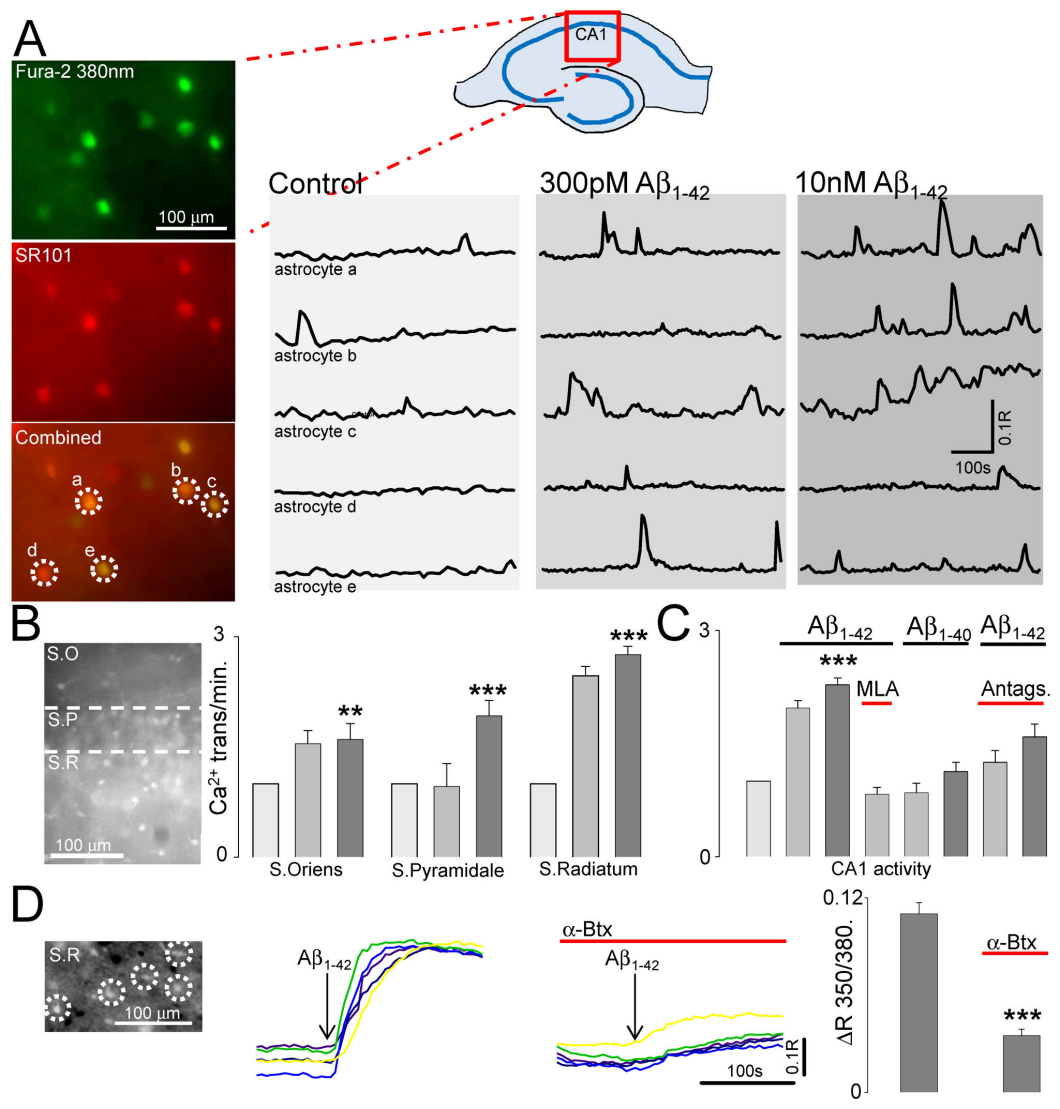
Aβ_1-42_ induces astrocytic calcium elevations. Center, image of hippocampal slice showing location of imaged CA1 area conducted in this study. **A.** Top left, Fluorescence image at 380nm of Fura-2 loaded slice. Middle image shows SR101 loading of astrocytes in same region. Combined image at bottom identifies co-loaded astrocytes. 350/380nm Ratio fluorescence traces over time for the circled astrocytes ‘a’ to ‘e’ are shown on the right, in control conditions, with 300pM Aβ_1-42_, and in the presence of 10nm Aβ_1-42_. **B.** Bargraph of astrocytic calcium transient frequency under control condition (light grey) and in response to 300pm (grey) or 10nM Aβ_1-42_ (dark grey) in CA1 Strata Oriens, Pyramidale and Radiatum. Data normalized to spontaneous activity in ACSF. **C.** Summary bargraph showing increased CA1astrocytic activity in response to Aβ_1-42_, which is inhibited by MLA and not affected by Aβ_1-40_. **D.** Left, fluorescent ratio image of a region of the Stratum Radiatum in CA1, with 4 circled astrocytes. 350/380 Ratio fluorescence traces from these astrocytes are shown to the right illustrating their response to focal 10nM Aβ_1-42_ application in the absence and presence of 100nM α-Btx. Bargraph on the right summarises the results from 4 such experiments.

We first determined the effect of Aβ_1-42_ bath application on astrocytic activity whilst monitoring astrocytic [Ca^2+^]_i_ transients in rat hippocampal slices. Concentrations of Aβ_1-42_ were used that are considered physiological (300pM) [22] in addition to that which would be expected to occur in the pathology of early AD (10nM) [23,24]. The ratiometric indicator Fura-2 was used so that extended imaging of the same astrocytes could be conducted whilst minimising confounding effects of bleaching. Slices were co-loaded with SR101 to allow identification of astrocytes loaded with Fura-2 [25] (Figure 1A). Analysis of [Ca^2+^]_i_ transients was restricted to SR101 positive cells. Astrocytes displayed spontaneous transients in the presence of TTX (0.044 ± 0.006 transients/min, n=115 astrocytes, 5 preparations) as reported previously for many brain areas, including hippocampus [17,26,27]. Astrocytic activity was increased by application of 300pM (0.087 ± 0.0085) and by 10nM Aβ_1-42_.(0.10 ± 0.009, n=115 astrocytes 5 preparations P<0.005) (Figure 1A). Astrocytic responses to Aβ_1-42_ did not follow a typical dose-response relationship, possibly due to α7nAChR desensitization properties as previously described [13,14]. Analysis of the effect in different CA1 strata showed that Aβ_1-42_ caused increased activity throughout CA1, with a greater effect being seen in stratum radiatum (175% increase to 10nM) (Figure 1B) where astrocytes ensheath afferent synapses [28]. The effect of Aβ_1-42_ was inhibited in the presence of the α7nAChR-selective antagonist methyl-lycaconitine (MLA) at 10nM (ctrl: 0.012 ± 0.017; Aβ_1-42_ 0.098 ± 0.0089, n=5) (Figure 1C), indicating involvement of this receptor. Bath application of Aβ_1-40_ did not lead to a significant increase in astrocytic activity (Ctrl: 0.0743 ± 0.0092, 300pM: 0.0629 ± 0.0076,10nM: 0.0838 ± 0.01, n=105 astrocytes 3 preparations), supporting a specific action of the Aβ_1-42_ peptide, and consistent with previous findings on Aβ–α7nAChR interactions [10,11,13]. To isolate, and confirm direct astrocytic location of the α7nAChR mediated effect we applied Aβ_1-42_ to slices in the presence of antagonists to the major hippocampal neurotransmitter receptors which might induce astrocytic [Ca^2+^]_i_ elevations (CPCCOEt, LY34195, MTEP, NBQX, D-APV, SR95531, CGP55845 and PPADS). Under these conditions Aβ_1-42_ still led to an increase in astrocytic activity, (ctrl: 0.052 ± 0.0071, 300pM Aβ_1-42_: 0.0652 ± 0.0083 10nM Aβ_1-42_: 0.083 ± 0.009, 113 astrocytes 5 preparations P<0.005), indicating that Aβ_1-42_ directly acts on astrocytic α7nAChRs. To investigate rapid agonist effects, Aβ_1-42_ was applied focally via a glass micropipette to the CA1 area. 10nM Aβ_1-42_ induced rapid [Ca^2+^]_i_ elevations in astrocytes in the area of application, which were inhibited by 100nM alpha bungarotoxin (Aβ_1-42_ ratio increase 0.11± 0.007; with α-Btx 0.035 ± 0.004, n=101 astrocytes, 4 preparations, P<0.0001) (Figure 1D). 

**Figure 2:**
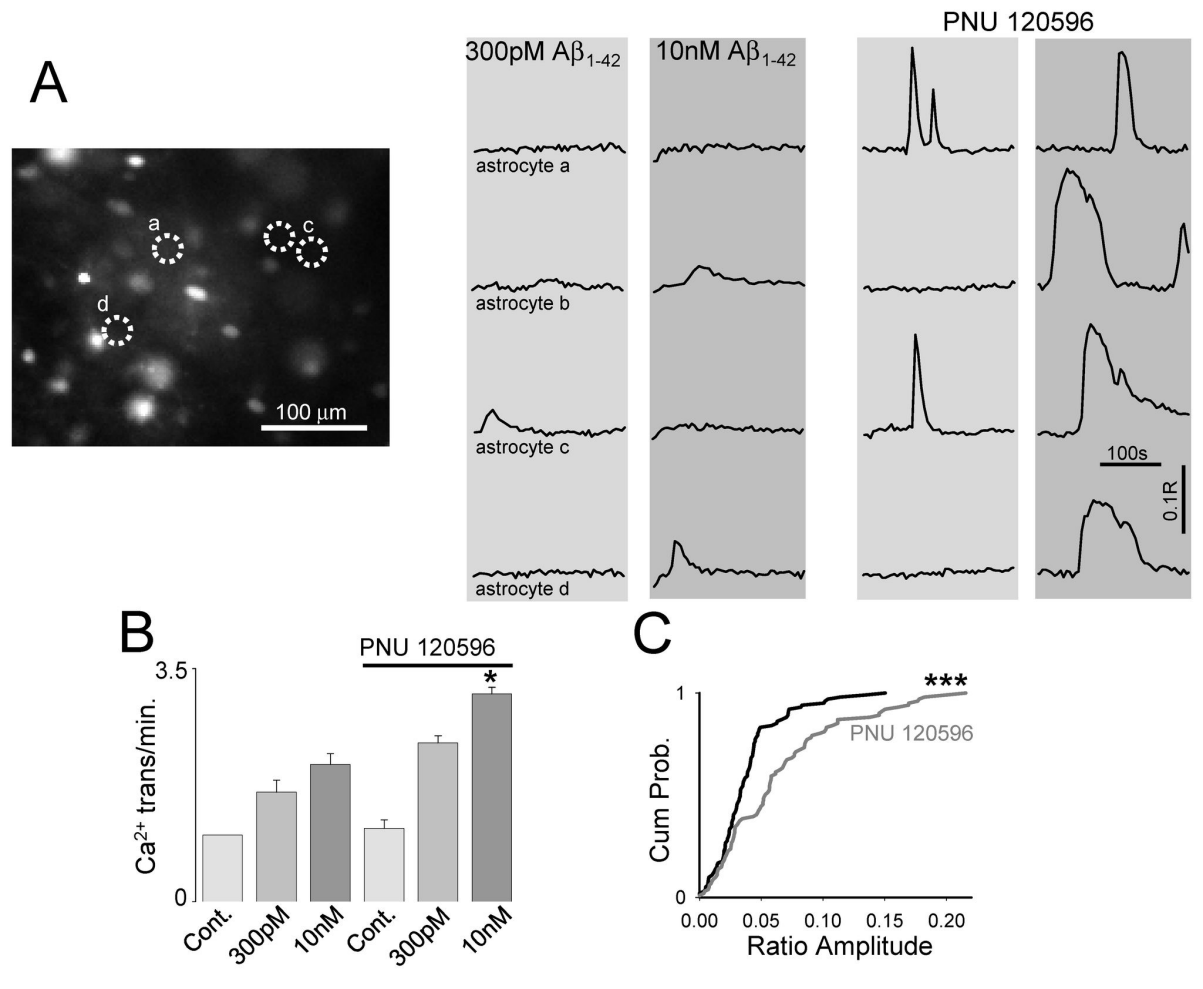
Aβ_1-42_-mediated astrocytic activation is α7nAChR mediated. **A.** Fluorescence ratio image of CA1 loaded with Fura-2AM indicating location of monitored astrocytes. Four astrocytes are circled, and ratio traces over time for these astrocytes are plotted to the right in the presence of Aβ_1-42_, and the α7nAChR allosteric modulator PNU120596 **B.** Bargraph summarising astrocytic activity frequency normalised to control ACSF. **C.** Cumulative distribution plot of calcium event ratio amplitude for 10nM Aβ_1-42_ with and without the presence of PNU120596.

To further investigate the mechanism and cellular action of Aβ_1-42_, we used the α7nAChR selective positive allosteric modulator PNU120596. Treatment with PNU120596 enhanced the response of astrocytes to Aβ_1-42_ (Figure 2A,B), increasing both astrocytic [Ca^2+^]_i_ transient frequency (300pM: 0.054 ± 0.0098, 10nM: 0.0693 ± 0.0115, following PNU120596 300pM 0.082 ± 0.009 10nM: 0.11 ± 0.01, P<0.05, 102 astrocytes 3 preparations) (Figure 2A,B) and the ratio of the individual [Ca^2+^]_i_ elevations (Figure 2C) (10nM Aβ_1-42_: 0.038 ± 0.0273 R, Aβ_1-42_ and PNU120596 0.064 ± 0.049. P<0.00001 K-S test). 

**Figure 3:**
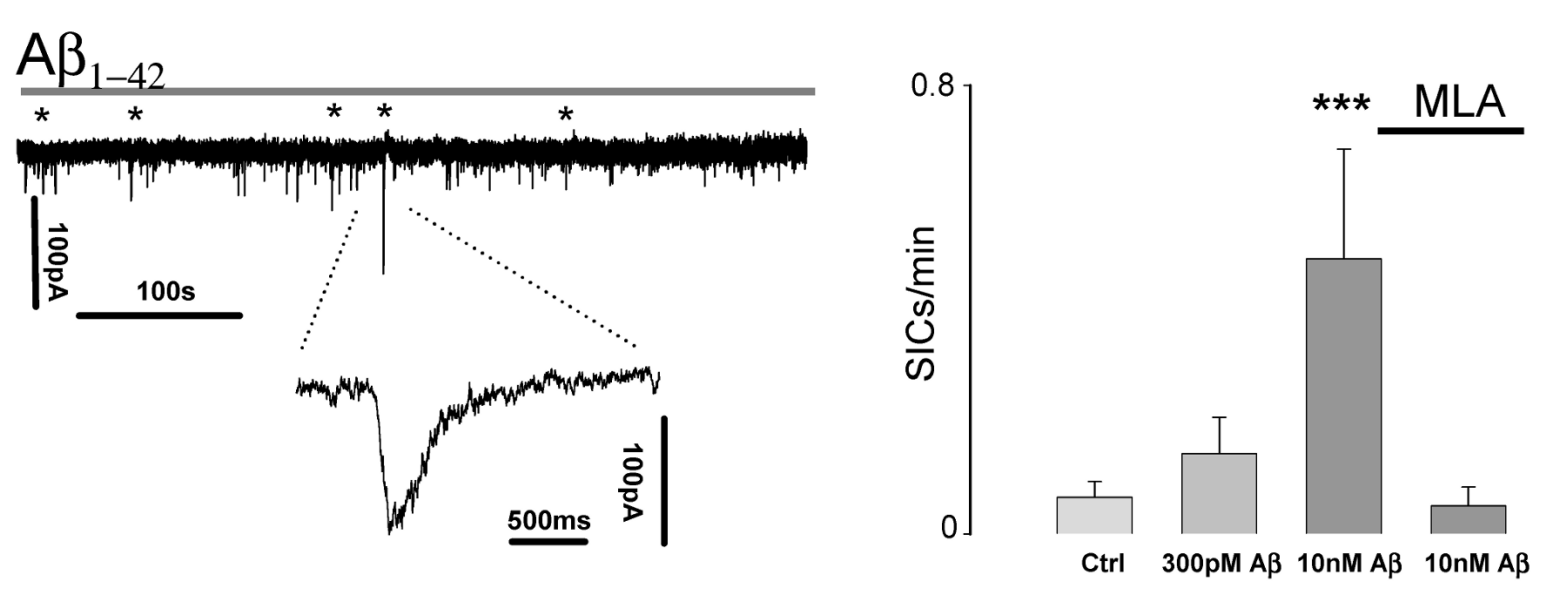
Aβ_1-42_ elicits astrocytic GT glutamate release. **A.** Patch clamp recording from CA1 pyramidal neuron showing elicitation of SICs following Aβ_1-42_ wash on. SICs marked with asterisks. **B.** Summary bargraph of SIC frequency increase in response to different concentrations of Aβ_1-42_ and their block by MLA.

Since it is well established that astrocytic [Ca^2+^]_i_ elevations can lead to GT release, [5,7,17,19,29], notably that of glutamate, the increase in astrocyte activity by Aβ_1-42_ may therefore also be predicted to cause an increase in slow inward currents (SICs) in hippocampal principal cells. We tested this mechanism by conducting patch clamp recordings from CA1 pyramidal neurons before and during Aβ_1-42_ wash on. Aβ_1-42_ application indeed induced SICs and led to an increase in their frequency following 10nM Aβ_1-42_ exposure (0.49 ± 0.2 SICs/min, n=6) compared to the preceding control period (0.065 ± 0.029 SICs/min, n=21, P<0.005). The induced increase was inhibited by the α7nAChR antagonist MLA (0.05 ± 0.034, n=6) (Figure 3), and blocked by 50∝M of the NMDA receptor antagonist D-APV (n=4), indicating that Aβ_1-42_ acts at α7nAChRs to result in astrocyte glutamate release that subsequently activates NMDARs (data not shown).

**Figure 4:**
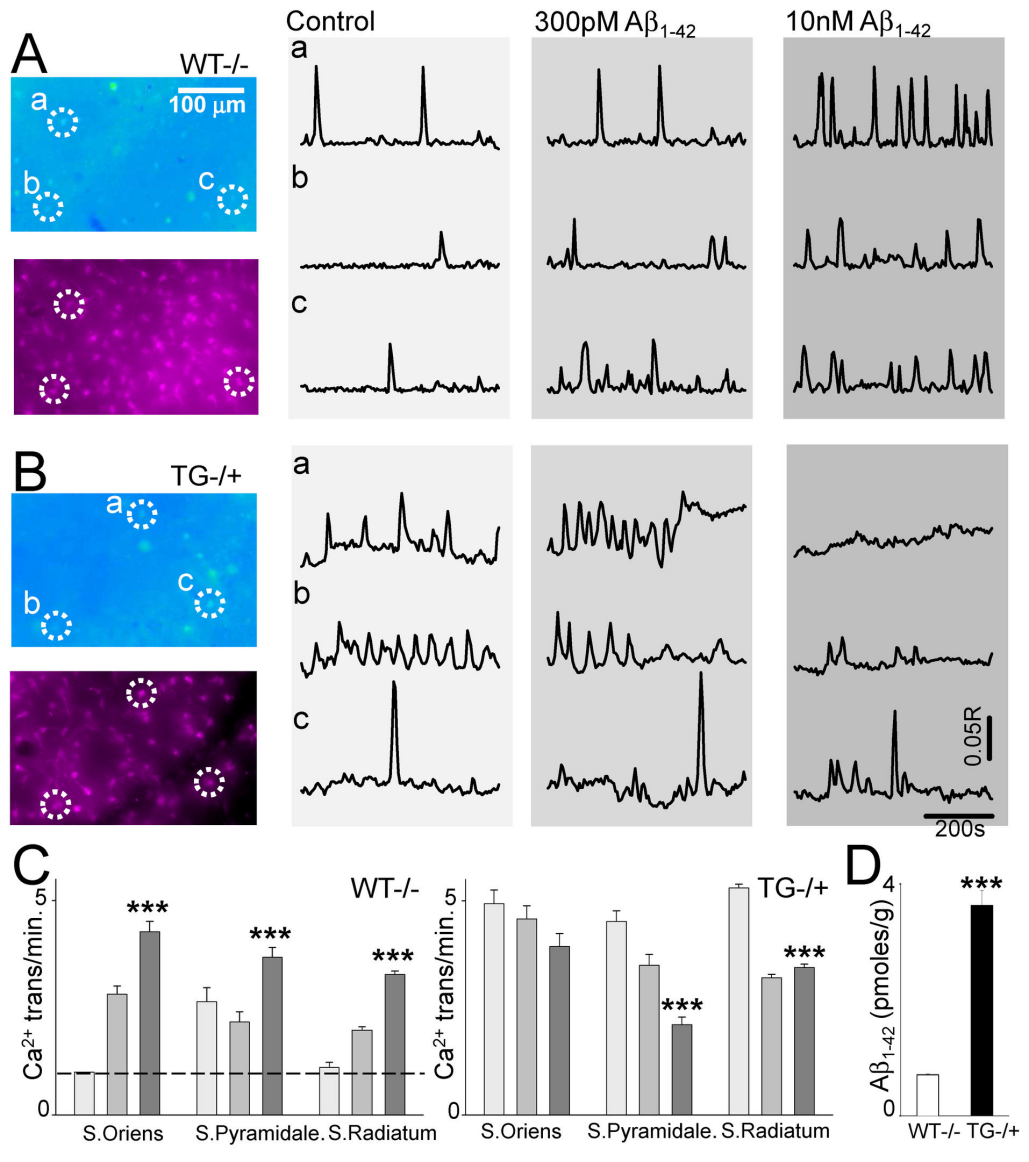
Differential effect of Aβ_1-42_ on astrocyte responses in WT and Tg2576 hippocampal slices. **A.** Top. Fura-2 340/380 ratio image of WT CA1 astrocytes. Below, red image of SR101 astrocyte loading of the same area with exemplar astrocytes ‘a’ to ‘c’ circled. Ratio traces from circled astrocytes are shown to the right in control ACSF, and with 300pM or 10nM Aβ_1-42_. **B.** Fura-2 ratio image of Tg2576 CA1 with SR101 labelling illustrated below. Ratio traces for the circled astrocytes under control condition and Aβ_1-42_ are shown to the right. **C.** Bargraphs summarising astrocytic transient frequency in CA1 strata from WT (left) and Tg2576 (right) mice, indicating an increased response to Aβ_1-42_ in WT but a reduction in Tg2576. **D.** Aβ_1-42_ levels in WT and TG2576 from P4,P14 determined with ELISA.

If Aβ_1-42_ exposure acts to increase astrocytic activity, we next asked what happens under conditions of chronic elevated Aβ *in vivo* as is likely the case in AD. Evidence from previous studies indicated that astrocytic activity is increased in an APP/PS1 AD model [30]. We therefore addressed the specific contribution of APP to astrocytic activity by using the Tg2576 AD mouse model that expresses a mutant form of human APP (K670→N and M671→L) resulting in Aβ overproduction and accumulation with concomitant synaptic and learning deficits [31,32]. In hippocampal slices from wild type littermates, addition of Aβ_1-42_ had similar effects to those observed in rat hippocampal slices, with Aβ_1-42_ increasing astrocyte calcium transients (Ctrl: 0.091 ± 0.01, 300pM 0.143 ± 0.009, 10nM 0.236 ± 0.016, n=7 preparations, P<0.001) (Figure 4A). In Tg2576 hippocampal slices, spontaneous astrocytic activity was elevated compared to wild type slices (Ctrl: 0.36 ± 0.028 vs. 0.091 ± 0.01, P<0.05). In contrast to wild type, wash on of Aβ_1-42_ to hippocampal slices from Tg2576 mice caused a significant decrease in calcium transients (300pM 0.23 ± 0.16, 10nM 0.23 ± 0.018, n=3 preparations, P<0.001) (Figure 4B). This effect was consistent across the CA1 hippocampal layers (Figure 4C). Since our hippocampal slices originate from very young mice, we confirmed that Aβ_1-42_ is aberrantly elevated in Tg2576. Thus, we determined Aβ_1-42_ levels in the brains of WT and Tg2576 animals at ages corresponding to the slice experiments (P4 and P14) and found that Aβ_1-42_ was significantly elevated in samples from Tg2576 (3.62 0.26pmoles/g, n=7) compared to WT (0.71 0.014pmoles/g, n=7, p<0.00001). Since we used a human Aβ_1-42_ ELISA, values in the WT represent background. Although we cannot rule out the effects of chronic exposure to additional products generated from APP processing on astrocytic function (e.g., APP C-terminal fragments [33]), the most parsimonious explanation of this and our imaging data thus far is that enhanced mutant APP expression *in vivo* resulting in aberrant Aβ production and accumulation, leads to increased astrocytic activity and alters the way that astrocytes subsequently respond to application of additional Aβ_1-42_.

**Figure 5:**
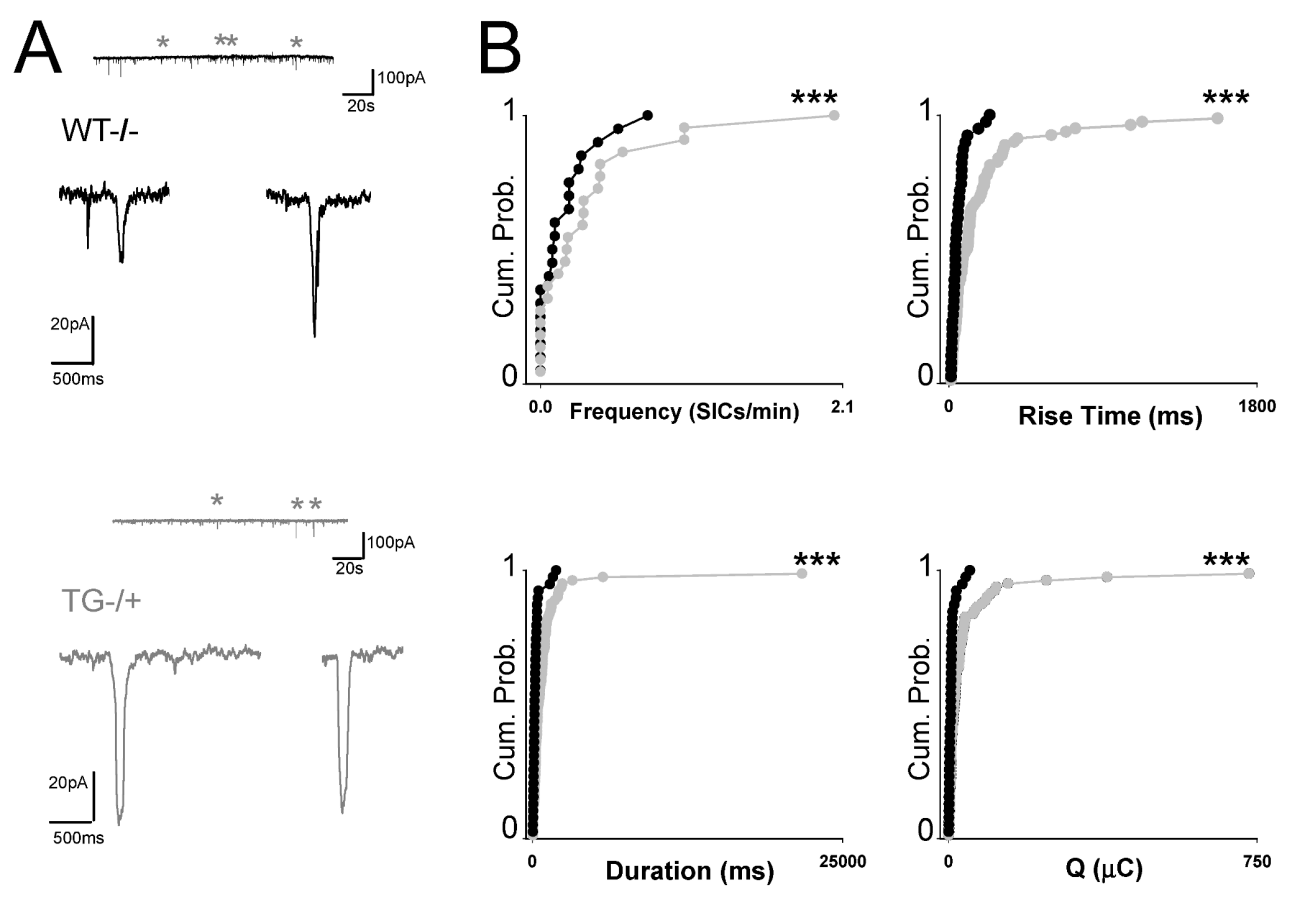
Spontaneous gliotransmission is altered in Tg2576. **A.** Patch clamp recording from a CA1 pyramidal neuron from a WT littermate, illustrating spontaneous SIC incidence. Two exemplar SICs are shown expanded beneath. Also shown is a recording from a Tg2576 hippocampal slice with expanded SICs illustrated beneath. **B.** Cumulative probability distributions of SIC parameters frequency, rise time, duration and charge (Q) for WT (black lines) and Tg2576 (dark grey lines).

**Figure 6:**
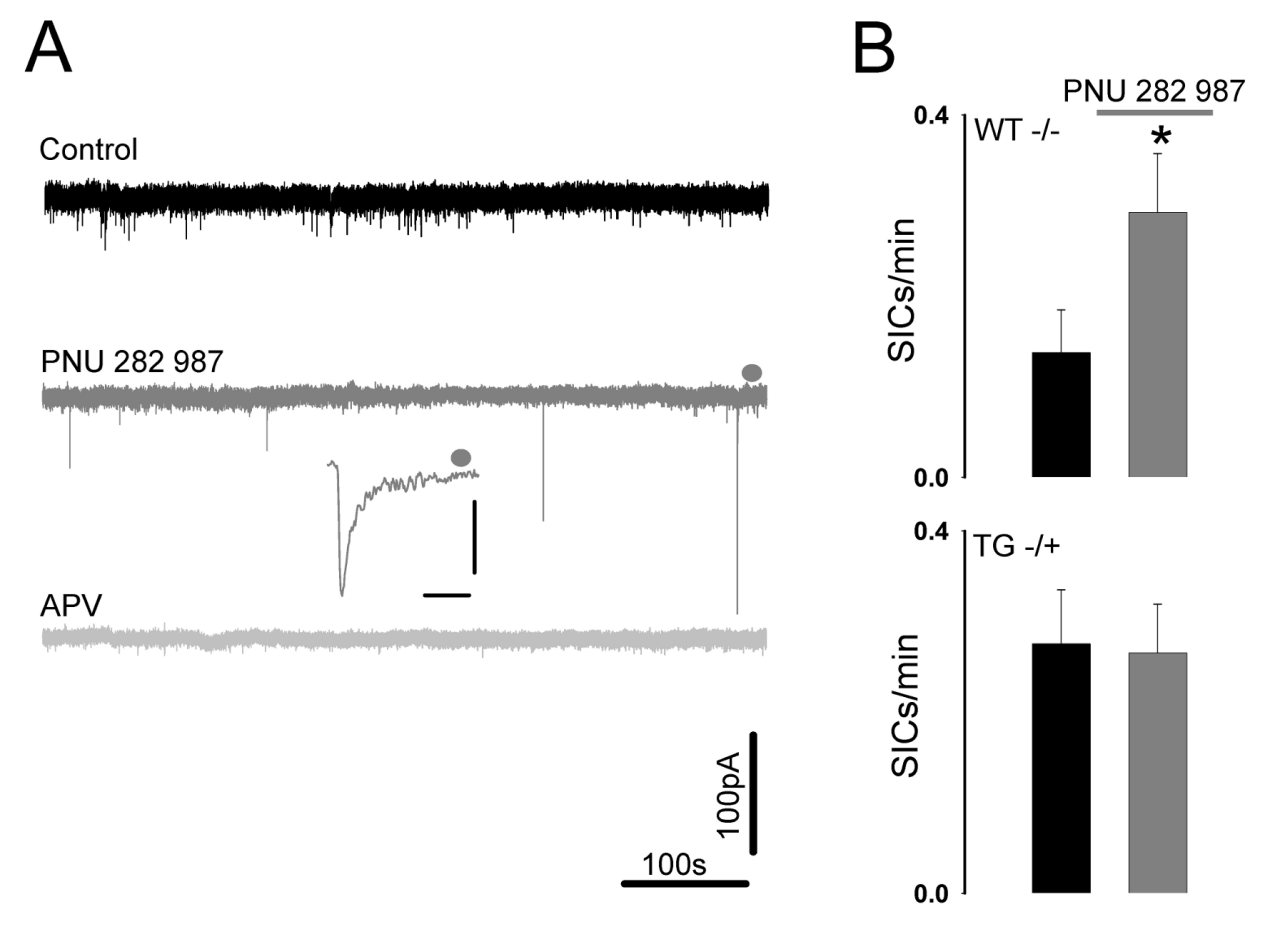
α7nAChR-mediated glutamate gliotransmission is abrogated in Tg2576. **A.** Patch clamp recording from a WT hippocampal CA1 neuron. Application of PNU 282987 induces SICs which are blocked by D-APV. **B.** Bargraphs summarising SIC frequency in WT and Tg2576 hippocampal slices following PNU 282987 wash on.

Aβ-induced increases in astrocytic activity and consequent astrocyte glutamate release might also be predicted to have an effect on SIC generation. We therefore recorded SICs from CA1 pyramidal neurons in slices from wild type and Tg2576 mice. Both genotypes exhibited SICs (Figure 5A); however, consistent with elevated spontaneous astrocytic activity, Tg2576 mice exhibited increased SIC frequency as well (0.16 ± 0.045 SICs/min, n=20; 0.34 ± 0.10 SICs/min, n=22, P<0.0001 K-S); possibly a result of α7 nAChR up-regulation previously reported for this AD model [34,35]. In addition, the kinetic properties of the recorded SICs also proved to be different in that SICs in Tg2576 pyramidal neurons had both longer rise times (182.11 ± 29.44ms, n=79 compared to 58.65 ± 8.24ms, n=39; for Tg2576 and wild type, respectively P<0.00001 K-S) and current duration (1047.01 ± 282.11ms, n=79 compared to 330.44 ± 66.41ms, n=39; for Tg2576 and wild type, respectively, P<0.00001 K-S) though similar amplitudes (72.8 ± 8.26pA n=79, compared to 66.01 ± 11.45pA, n=39; for Tg2576 and wild type, respectively) culminating in Tg2576 CA1 pyramidal neuron SICs conferring a greater charge transfer (40.96 ± 10.8.∝C, n=79) than wild type (8.85±1.9 µC, n=39, P<0.0001 K-S) (Figure 5D). The observed differences in the response of hippocampal astrocytes to Aβ_1-42_ application in slices from wild type and Tg2576 mice may be an effect of *in vivo* Aβ exposure on α7nAChR-mediated GT release pathway. To more directly test this, we used a selective α7nAChR agonist as a tool to induce astrocytic glutamate release and resultant SICs. Patch-clamp recordings from wild type CA1 pyramidal neurons following perfusion of 30µM PNU 282987 resulted in an increase in SIC frequency (ctrl: 0.1379 ± 0.047 SICs/min, agonist: 0.29 ± 0.065 SICs/min n=7, P<0.05) (Figure 6) whilst in neurons recorded from Tg2576 hippocampus, the agonist had no overall further effect on the already elevated SIC frequency (ctrl: 0.275 ± 0.060 SICs/min, agonist: 0.265 ± 0.053 SICs/min n= 9,) (Figure 6B). In individual experiments the difference was manifested by a general increase in WT SIC frequency but a mixture of responses in Tg2576 slices consistent with dysregulated [Ca^2+^]_i_ – GT release pathways.

## Discussion

In this study we demonstrate a role for Aβ having an impact on hippocampal neurons via interaction with astrocytic α7nAChR, a Ca^2+^-permeable ligand gated receptor. Furthermore, we provide the first description of hippocampal astrocyte dysfunction in anatomically intact acute slices from the Tg2576 mouse model for AD-like amyloidosis. Since we observed these alterations in hippocampal slices from very young animals, our data suggest that hippocampal astrocytes represent a particularly vulnerable target in AD prior to overt deficits in hippocampal plasticity and cognition [34,36,37]. 

### Aβ activates α7nAChRs at physiologically relevant concentrations

Aβ binds and activates α7nAChRs at picomolar concentrations [10,11,13,38,39]. Our previous work [13] showed that picomolar concentrations of Aβ_1-42_ had agonist effects on α7nAChRs expressed in *Xenopus* oocytes while at higher (nanomolar) concentrations acted as an antagonist. Subsequent studies by other groups stirred debate as some confirmed agonist properties [22,39] whilst others found antagonist effects [40,41]. Discrepancies may be partly explained by the dynamic nature of Aβ and its structural sensitivity to pH, osmolarity, temperature, and concentration, since methods of preparation vary between groups and aggregation status is not always confirmed; indeed different effects due to concentration, and aggregation state have been reported within some studies [12,13,18,22,39]. In the present study we used a method which produces soluble oligomeric Aβ_1-42_ predominantly comprised of hexamers [12,13,18,37]. 

We found that picomolar Aβ_1-42_ induced [Ca^2+^]_i_ elevations in hippocampal CA1 astrocytes, which were inhibited by an α7nAChR-selective antagonist and enhanced by an α7nAChR-specific positive allosteric modulator. Our results indicate that bath application of α7nAChR ligands, including Aβ_1-42_, resulted in activation of at least a subset of the α7nAChR receptor population so that we were able to observe astrocyte responses that continued over hundreds of seconds. Our results are consistent with prolonged Aβ_1-42_-mediated [Ca^2+^]_i_ signals elicited by α7nAChR activation in astrocytes [16] and presynaptic terminals [39] and likely also involve a contribution from intracellular calcium release [15,42].

Focal application of Aβ resulted in direct and rapid increases in astrocyte calcium consistent with a direct agonist, while bath application did not usually result in rapid synchronised calcium elevations but rather in an increase in frequency of astrocyte calcium elevations. Because of the described desensitising action of α7nAChRs it is intriguing that such a prolonged increase is seen, however it must be noted that there is little information on astrocyte α7nAChRs, or how rapidly they recover from desensitisation. Alternatively, it may also be that the low Aβ concentrations used in our preparation are able to interact dynamically with the α7 nAChR over a prolonged period, or that α7nAChR interaction with CICR (as shown in culture by Sharma and Vijayraghavan [15]) results in establishment of long lasting increase in calcium elevation frequency.

In addition to their expression on primary neurons and interneurons in the hippocampus [43], functional α7nAChRs are also expressed on NG2+ glial cells [44]. While this could potentially confound the deciphering of experimental results, our approach of blocking action potential propagation and utilizing neurotransmitter receptor antagonists to block secondary effects of neuronal α7nAChR activation indicate that there is direct astrocytic α7nAChR activation by Aβ_1-42_. In the absence of antagonists the astrocytic response to Aβ_1-42_ was greater, indicating either a component effect on presynaptic α7nAChR leading to additional astrocytic excitation or that α7nAChR induced GT release results in astrocytic paracrine signalling that amplifies the Aβ_1-42_ effect. Both these scenarios may be important for the pathological roles of astrocytes in AD. 

There is currently great interest and debate surrounding the relationship between astrocyte receptor activation, astrocytic [Ca^2+^]_i_ elevation and GT release. Some findings support the interpretation that modifying Gq G-protein-coupled Ca^2+^ release fails to impinge upon synaptic transmission and LTP induction [45] whilst others indicate that specific [Ca^2+^]_i_ elevation patterns caused by different agonists induce neuronal SICs which can influence plasticity [46]. Astrocytes in some brain areas, including hippocampus, also express ligand gated ion channels such as NMDA and P_2X_ receptors [47], as well as α7nAChRs [15,16]. Activation of such channels at the negative astrocytic resting membrane potential would be expected to create a significant [Ca^2+^]_i_ signal due to the favourable calcium electrochemical gradient and the high Ca^2+^ permeability of α7nAChRs. In this study we found that [Ca^2+^]_i_ elevations were increased following Aβ_1-42_ exposure and enhanced by the action of an α7nAChR allosteric modulator PNU 120596. 

Activation of the ligand gated ionotropic receptor α7nAChR by Aβ_1-42_ as well as by specific α7nAChR agonists also resulted in SICs. Therefore, in addition to G-protein coupled receptor activation, astrocytic hippocampal ligand gated α7nAChR activation also induces gliotransmission. 

### Astrocytic activity is dysregulated in an animal model for AD

Recording SICs in CA1 neurons enabled us to determine that the observed effects on astrocytic [Ca^2+^]_i_ had effects on GT release as well as additionally demonstrate that SICs were different in Tg2576 compared to WT littermates that resulted in enhanced impact of NMDA-mediated currents on CA1 neurons. Interestingly, while Aβ_1-42_ increased [Ca^2+^]_i_ transients in WT mice, [Ca^2+^]_i_ transient frequency was not further increased in Tg2576 mice. Directly probing α7nAChR mediated astrocytic gliotransmission demonstrated that this difference was also reflected in α7 nAChR function since activation with a selective α7nAChR agonist induced SICs in slices obtained from WT littermates but not in Tg2576. This difference in the α7nAChR-mediated GT pathway may reflect specific changes in α7nAChR function or a multi-faceted dysregulation in GT release regulation; the latter scenario being supported by the observed increased spontaneous [Ca^2+^]_i_ elevations and GT release from Tg2576 astrocytes. 

Our observation that astrocytic [Ca^2+^]_i_ transient frequency in Tg2576 mice was increased compared to WT littermates agrees with *in vivo* astrocytic imaging studies on an APP/PS1 transgenic model of AD [30] in which spontaneous cortical astrocytic [Ca^2+^]_i_ transients and synchrony in the vicinity of plaques was increased. Since we interrogated slices obtained from APP mice at a much younger age suggests that soluble Aβ also has effects on these parameters; although we cannot rule out the contribution of additional APP metabolites [33]. Importantly our study shows that Aβ_1-42_ levels are elevated in the first two postnatal weeks, long before synaptic and cognitive deficits can be detected [13,36,37] at 5-6 months and coincide with insoluble Aβ_1-42_ deposition [48,49]. Thus, increased [Ca^2+^]_i_ transients in Tg2576 hippocampal astrocytes indicates that astrocytic function can be affected during very early disease processes. 

### Functional implications of astrocytic α7nAChRs

Our finding that Aβ_1-42_-α7nAChR interaction increases astrocytic activity to induce glutamate gliotransmission has several implications regarding our understanding of astrocyte-neuron interaction in the hippocampus. Activation of hippocampal α7nAChRs contributes to synaptic plasticity and cognition [50-53], and it has been previously reported that physiological levels of Aβ_1-42_, acting via α7nAChR receptors, can enhance the induction of hippocampal LTP [22]. Our data, together with the increasing realization of GT influence on different forms of hippocampal synaptic plasticity [2,6,7], posits a role for astrocytic α7nAChR-mediated GT release as a potential physiologically relevant mechanism in hippocampal synaptic plasticity, especially considering synaptic activity-induced Aβ release [9].

In AD there is accumulation of Aβ in the brain leading to cognitive impairment hypothesized to initially be due to synaptic failure [54,55]. Within such a context, Aβ-mediated dysregulation of gliotransmission would be expected to disrupt the intermediary role of astrocytes in synaptic function, while increased glutamate from enhanced gliotransmission might also lead to increased excitability within the hippocampal neural network. It has been proposed that elevated Aβ_1-42_ inhibits LTP induction by increasing extra-synaptic NR2B subunit-containing NMDA receptors leading to aberrant ERK MAPK activation [56]. Glutamate transporters and an increase in extra-synaptic spillover were implicated, while selectively inhibiting glutamate uptake mimicked the effects of Aβ_1-42_ on extrasynaptic NMDA receptors and increased MAPK activation. Since inhibiting glutamate transporters in the hippocampus increases astrocytic glutamate release [57] via NR2B-containing receptors and we previously showed that activation of α7nAChRs by Aβ_1-42_ increases hippocampal ERK MAPK activity [12,18]. Therefore, from our data, one might predict that elements of the MAPK pathway may be recruited by Aβ via astrocytic α7nAChR-induced gliotransmission in AD. Interestingly, Tg2576 mice exhibit age-dependent cognitive decline as measured in several behavioral paradigms, but most notably in those requiring proper hippocampal MAPK function which are also impaired in human AD [37,49,58,59]. In human AD and mouse models of the disease, astrocytes are known to become activated, participate in the brain inflammatory response [60-62] and undergo morphological changes [63]. 

A contemporaneous study found that an α-BTx sensitive Aβ_1-42_-α7nAChR interaction resulted in sustained glutamate release from cultured astrocytes [64], and that ambient glutamate levels, detected using *in vivo* microdialysis, were α7nAChR dependent. Our study, focusing on the frequency and kinetics of SICs which result from phasic astrocytic glutamate release as a result of Aβ_1-42_-α7nAChR interaction, provides insight into the dynamic mechanistic relationship between Aβ and GT release in the anatomically intact hippocampal slice.

It is striking that we observed such early changes in astrocytic function in Tg2576, at an age at which cognitive and synaptic function are unaltered since studies on 3-4 months-old Tg2576 reported no synaptic or cognitive deficits [36,37]. Our results therefore indicate that astrocyte dysfunction could be an important player in preclinical AD. Thus, if possible, monitoring early astrocyte [Ca^2+^]_i_ activity and GT release could serve as an important biomarker for AD and presage later AD-related cognitive impairment. Our findings additionally identify astrocytic α7nAChRs as potential therapeutic targets. 
